# Prevention of Autoimmune Diabetes in NOD Mice by Dimethyl Fumarate

**DOI:** 10.3390/antiox10020193

**Published:** 2021-01-29

**Authors:** Shiri Li, Nosratola D. Vaziri, Lourdes Swentek, Chie Takasu, Kelly Vo, Michael J. Stamos, Camillo Ricordi, Hirohito Ichii

**Affiliations:** 1 Department of Surgery, University of California, Irvine, CA 92868, USA; lyrobles@hs.uci.edu (L.S.); takasu.chie@tokushima-u.ac.jp (C.T.); kmvo89@gmail.com (K.V.); mstamos@hs.uci.edu (M.J.S.); 2 Department of Medicine, University of California, Irvine, CA 92868, USA; ndvaziri@hs.uci.edu; 3 Cell Transplant Center, Diabetes Research Institute, University of Miami, Miami, FL 33136, USA; CRicordi@med.miami.edu

**Keywords:** diabetes, non-obese diabetic mice, Nrf2, dimethyl fumarate, antioxidant, oxidative stress

## Abstract

Oxidative stress plays critical roles in the pathogenesis of diabetes. This study tested the hypothesis that by protecting β-cells against oxidative stress and inflammation, an Nrf2 activator, dimethyl fumarate (DMF), may prevent or delay the onset of type 1 diabetes in non-obese diabetic (NOD) mice. Firstly, islet isolation was conducted to confirm the antioxidative effects of DMF oral administration on islet cells. Secondly, in a spontaneous diabetes model, DMF (25 mg/kg) was fed to mice once daily starting at the age of 8 weeks up to the age of 22 weeks. In a cyclophosphamide-induced accelerated diabetes model, DMF (25 mg/kg) was fed to mice twice daily for 2 weeks. In the islet isolation study, DMF administration improved the isolation yield, attenuated oxidative stress and enhanced GCLC and NQO1 expression in the islets. In the spontaneous model, DMF significantly reduced the onset of diabetes compared to the control group (25% vs. 54.2%). In the accelerated model, DMF reduced the onset of diabetes from 58.3% to 16.7%. The insulitis score in the islets of the DMF treatment group (1.6 ± 0.32) was significantly lower than in the control group (3.47 ± 0.21). The serum IL-1α, IL-1β, IL-2, IL-4, IL-5, IL-6, IL-9, IL-12p70, IFN-γ, TNF-α, MCP-1 and CXCL16 levels in the DMF-treated group were lower than in the control group. In conclusion, DMF may protect islet cells and reduce the incidence of autoimmune diabetes in NOD mice by attenuating insulitis and proinflammatory cytokine production.

## 1. Introduction

It is generally accepted that reactive oxygen species (ROS) contribute to the autoimmune-mediated destruction of pancreatic β-cells in the islets of Langerhans and loss of insulin secretion [1]. Antigen-specific T cells mediate the infiltration of inflammatory cells into the pancreas, which leads to the production of inflammatory cytokines and ROS and eventually promotes β-cell destruction. It is well known that innate immune cells, such as macrophages and dendritic cells, are the first cells that enter the islets during insulitis [2,3]. The ROS generated by the initial insult to the islets induce the activation of macrophages, redox-dependent NF-kB and other transcription factors [4]. Activated macrophages secrete a mixture of proinflammatory cytokines such as TNF-α, IL-6 and IL-1β, and produce ROS, which can damage the pancreatic β-cells [5,6,7]. Moreover, the activation of macrophages and T cells triggers oxidative stress, which leads to the progression of type I diabetes [8]. In addition, pancreatic β-cells contain very low levels of antioxidant enzymes, such as glutathione peroxidase, superoxide dismutase and catalase, which renders pancreatic islets highly susceptible to oxidative stress. The divergent antioxidant capacity of human islet cell subsets represents a potential cause of β-cell vulnerability in diabetes and islet transplantation [9].

ROS are a major factor in causing islet damage and loss during the islet isolation and transplantation processes. We previously demonstrated that one of the nuclear erythroid 2 related factor 2 (Nrf2) activators, dh404, could protect islets by promoting the nuclear translocation of Nrf2 and upregulating HO-1 in islet cells during the islet isolation procedure [10]. Moreover, a recent study by Yagishita Y et al. demonstrated that the activation of Nrf2 signaling is tightly associated with the prevention of autoimmune diabetes in non-obese diabetic (NOD) mice [11]. Dimethyl fumarate (DMF) was approved by the Food and Drug Administration and has been used for reducing the relapse rate and slowing the progression in patients with multiple sclerosis. The exact mechanism of action of DMF has yet to be clearly determined; however, it has been reported that DMF activates the Nrf2/Kelch-like ECH-associated protein 1 (Keap1) antioxidant pathway, elevates antioxidants and attenuates oxidative stress and inflammation [12,13]. Our previous study has also shown that DMF could protect human pancreatic islet cells by upregulating the expression of antioxidant molecules in vitro and improve islet function in L-arginine-induced chronic pancreatitis in rats [14,15]. We set out to measure the levels of expression of two genes encoding enzymes involved in redox homeostasis and cell defense against oxidative stress, i.e., glutamate-cysteine ligase catalytic subunit (GCLC), which is involved in step 1 of the biosynthesis of glutathione from L-cysteine and L-glutamate, and NAD(P)H quinone dehydrogenase 1 (NQO1), which serves as a quinone reductase in connection with conjugation reactions of hydroquinones involved in detoxification pathways and biosynthetic processes.

The NOD mouse provided the opportunity to assess a therapeutic intervention aimed at preventing or halting autoimmune-mediated β-cell loss in an animal model of type I diabetes. In this study, we first demonstrated the cytoprotective effects of DMF (oral administration) on islet cells through the elevation of GCLC and NQO1 in the rat islet isolation model. We then confirmed the efficacy of DMF treatment in minimizing insulitis and deferring the onset of diabetes in the NOD mouse model of type 1 diabetes (spontaneous and accelerated diabetes models) through the upregulation of cellular antioxidant and anti-inflammatory machinery.

## 2. Materials and Methods

### 2.1. Animals

Eight-week-old male Sprague-Dawley (250 g) rats were purchased from the Charles River company (Wilmington, MA, USA), and eight-week-old female NOD mice were purchased from the Jackson Laboratory (Bar Harbor, Maine). All the animals were free of pathogens and housed under standard conditions (room temperature, 22 °C; humidity, 50 ± 5%; 12:12 h light/dark cycle). The study was approved by the Institutional Animal Care and Use Committee of the University of California, Irvine (#2011-3010 and #2012-3065). Stock solutions of DMF (Sigma, St. Louis, MO, USA) were dissolved in 0.08% methyl cellulose (Sigma, St. Louis, MO, USA) and given to rats and mice (25 mg/kg) by oral gavage. In the control group, the same amount of methyl cellulose was fed as vehicle by oral gavage [15].

### 2.2. Experimental Design

A rat islet isolation model was first used to investigate whether DMF could protect islets and improve islet yield against the ROS created during the islet isolation procedure, to confirm the antioxidative effect of DMF on islet cells. In the rat islet isolation study, rats were randomly divided into vehicle (*n* = 5) and DMF-treated (*n* = 5) groups. The DMF-treated rats received DMF (25 mg/kg, twice/day) by oral gavage for 2 days prior to the isolation.

In the NOD mouse study, the onset of spontaneous autoimmune diabetes was evaluated in female NOD mice. The mice were randomly divided into DMF-treated (*n* = 24) and control (*n* = 19) groups. DMF (25 mg/kg) or vehicle was given to the mice once daily starting at the age of 8 weeks through the age of 22 weeks in the DMF-treated group or the control group.

To investigate if DMF could reverse the hyperglycemia in the NOD mice with spontaneous diabetes, 6 mice with spontaneous diabetes were given DMF (25 mg/kg) once daily from week 22 for 6 weeks. In addition, to confirm the effect of DMF on the prevention of diabetes, DMF was discontinued in mice without spontaneous diabetes in the DMF treatment group at the age of 22 weeks. Those mice were monitored for another 6 weeks after the discontinuation of DMF administration.

In the accelerated diabetes model, 8-week-old female NOD mice were given a single dose of cyclophosphamide (20.0 mg/kg, Sigma, St. Louis, MO, USA) intraperitoneally after the confirmation of no hyperglycemia. The mice were randomly divided into DMF-treated (*n* = 12) and control (*n* = 12) groups. In the DMF-treated group, DMF was given to the mice (25 mg/kg) by oral gavage 12 and 24 h prior to cyclophosphamide administration, and continued twice daily for 2 weeks. Two weeks after cyclophosphamide injection, all the animals were euthanized for tissue and blood harvest. Daily blood glucose levels were monitored throughout the experiment.

### 2.3. Rat Islet Isolation

As described previously [10], rat pancreas was distended and digested with collagenase type V (Sigma-Aldrich, St. Louis, MO, USA), and the islets were purified using a Ficoll density gradient (Mediatech, Inc., Manassas, VA, USA). The islets were then collected and washed in HBSS. The crude number of islets in each diameter class was determined by counting after diphenylthiocarbazone (DTZ, MP Biomedicals, Santa Ana, CA, USA) staining. This number was then converted to the standard number of islet equivalents (IEQ; diameter standardizing to 150 um).

### 2.4. Quantitative Real-Time Reverse Transcription–PCR for Rat Islets

After islet isolation, the total RNA of the islets was isolated using TRIzol Reagent (Sigma-Aldrich) from approximately 300 islets according to the manufacturer’s instructions. The resultant DNA-free RNA samples were stored at −80 °C until use. The abundance of mRNA encoding GCLC and NQO1 was measured by quantitative real-time PCR using the Qiagen Quantitect SYBR Green RT-PCR system. Single-stranded complementary DNA (cDNA) was synthesized from 1 ug of total RNA using the Reverse Transcription System kit (Promega, Madison, WI). Real-time reverse transcription–PCR was performed as previously described [16,17]. The PCR conditions were as follows: 95 °C for 5 min; 40 cycles of 30 s at 95 °C, 30 s at 68 °C, and 60 s at 72 °C. The relative fold change compared with control was calculated using the comparative Ct method [18]. Ct is the cycle number at which the fluorescence intensity first exceeds the threshold level. ΔCt is Ct (target gene) _ Ct (internal control, GAPDH). The GCLC- and NQO1-specific primer sequences (Qiagen, Germantown, MD) are available upon request. The primer set for each gene was as follows. GCLC (accession number: NM_012815.2), Forward: 5′-CTGCACATCTACCACGCAGTCA-3′; Reverse: 5′- ATCGCCGCCATTCAGTAACAA-3′. NQO1 (accession number: NM_017000.3), Forward: 5′-TGGAAGCTGCAGACCTGGTG-3′; Reverse: 5′-CCCTTGTCATACATGGTGGCATAC-3′. Specificity of amplification products were verified by melting curve.

### 2.5. Immunofluorescence and Confocal Microscopy

8-hydroxy-2′-deoxyguanosine (8-OHdG) is an oxidation byproduct of deoxyguanosine. 8-OHdG is one of the most sensitive biomarkers of oxidative stress-related DNA injury to the nuclei or mitochondria, and has been widely used to assess oxidative stress. A total of 500–600 rat islets were dissociated with a previously reported method [9,10]. Briefly, the islets were incubated in 1 mL of pre-warmed Accutase solution (Sigma-Aldrich) at 37 °C for 10 min. Then, cold fetal bovine serum (Sigma-Aldrich) was added to stop the digestion. After washing with PBS, the dissociated single cells from rat islets were fixed on glass slides with 2.5% paraformaldehyde (Electron Microscopy Sciences, Washington, PA, USA). To minimize the nonspecific antibody binding, the fixed cells were incubated with Protein Block (BioGenex, San Ramon, CA, USA) for 1 h at room temperature. After incubation with proteinase K (10 μg/mL) for 7 min at room temperature, the slides were incubated for 2 h with mouse monoclonal anti-8-OHdG antibody (1:100, Abcam Cambridge, MA, USA). They were then washed and incubated with AlexaFluor-488 goat anti-mouse IgG (1:250; (Thermo Fisher Scientific, Waltham, MA, USA) and DAPI at room temperature for 90 min. The images were analyzed with a confocal microscope. At least 500 cells were evaluated to calculate the percentage of 8-OHdG-positive cells in each sample.

### 2.6. NOD Mouse Diabetes Monitoring

The animals were monitored daily for blood glucose levels, obtained via tail vein pricks using a contour glucometer (Bayer Healthcare, Mishakawa, IN, USA). Diabetes was defined as non-fasting glycemic values of ≥300 mg/dl on two consecutive readings.

### 2.7. Histopathology

In the accelerated diabetes model, mouse pancreas was collected and fixed in 10% buffered formalin solution, embedded in paraffin blocks, and 4 um-thick sectioned (five per pancreas). The pancreas tissue was processed for hematoxylin–eosin (H&E) staining using standard techniques. A score of 0 to 4 was assigned based on islet infiltration by two blinded experienced pathologists. The insulitis scores were graded as follows: grade 0, normal islets; grade 1, mild mononuclear infiltration (<25%) at the periphery; grade 2, 25–50% of the islets infiltrated; grade 3, >50% of the islets infiltrated; grade 4, islets completely infiltrated with no residual parenchyma remaining [19]. At least 30 islets per group were analyzed and pooled from sections obtained from different mice. 

### 2.8. Serum Cytokine Determination

Whole blood was obtained from anesthetized mice via cardiac puncture just prior to euthanasia. The blood was then centrifuged (3000 rpm for 20 min), and the supernatant, collected and analyzed using the mouse cytokine antibody array 3 (RayBiotech, Norcross, GA, USA), containing 62 murine cytokine-specific antibodies. The supernatant was placed on special membranes and blocked and washed several times, followed by the addition of a biotin conjugate anti-cytokine mixture and incubation at room temperature for 2 h. This was followed by the addition of conjugated horseradish peroxidase-labeled streptavidin (HRP-SA) and incubation at room temperature for 2 h. The membranes were washed and processed with Detection buffer and exposed with X-ray Film (Kodak, Rochester, NY, USA) [15,20].

### 2.9. Statistical Analysis

The data are expressed as mean ± standard deviation (SD), and the statistical differences between groups were determined by an unpaired *t*-test. The percentages of the onset of diabetes between two groups were compared using the log-rank (Mantel–Cox) test. *p* values < 0.05 were considered significant.

## 3. Results

### 3.1. DMF Administration Improved Isolation Yield, Attenuated Oxidative Stress and Enhanced GCLC and NQO1 Expression in Pancreatic Islets

**Figure 1:**
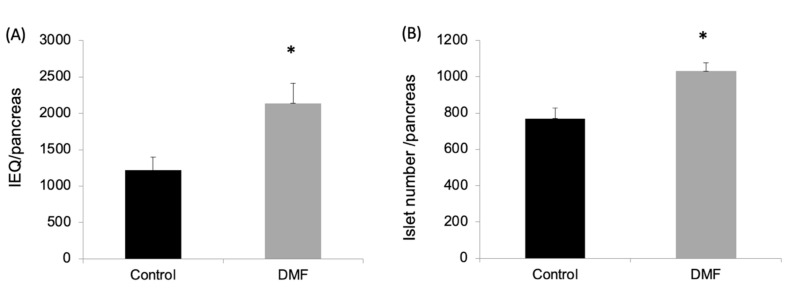
Dimethyl fumarate (DMF) treatment improved islet yield from the rats’ pancreases. After islet isolation, the islet yields (both islet equivalents (IEQ) (**A**) and islet number (**B**)) from the rat pancreases were significantly higher in the DMF-treated group than the control group (* *p* < 0.05, respectively).

**Figure 2:**
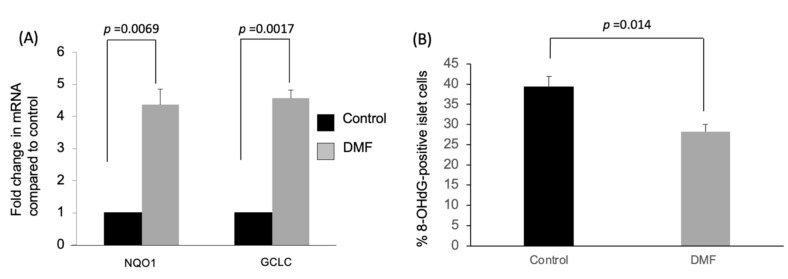
Impact of treatment with DMF on antioxidant enzymes and oxidative damage in isolated islet cells. (**A**) Effects of DMF administration on mRNA levels of antioxidant enzymes in the isolated islets were evaluated by qRT-PCR. Both GCLC and NQO1 mRNA expression of the isolated islets (*n* = 3 each group) in the DMF-treated group were four-fold higher compared to control group. (**B**) Immunofluorescent staining of the 8-OHdG was performed to evaluate oxidative stress in dissociated islet cells. 8-OHdG-positive islet cells (*n* = 4 for each group) were significantly decreased in the DMF-treated group compared to the control group (*p* = 0.014).

As shown in Figure 1A,B, both the islet yield and count were significantly higher in the DMF-treated group when compared to the vehicle-treated group (IEQ: 2136 ± 620 vs. 1231 ± 468; IC: 1032 ± 45 vs. 770 ± 57; *p* < 0.05). This was associated with a four-fold increase in the mRNA expression of the antioxidant enzymes GCLC and NQO1 in the DMF-treated group (Figure 2A). GCLC and NQO1 have been reported as putative transcriptional targets of Nrf2 and upregulated by cellular stress.

Immunofluorescent staining with 8-OHdG antibody was used to measure the effect of oxidative damage to DNA on the isolated islet cells. As shown in Figure 2B, the 8-OHdG-positive islet cells in the DMF-treated group were significantly lower when compared to the control group (28.3 ± 3.6% vs. 39.3 ± 5.3%, *p* = 0.014). Those results suggest that DMF increased the islet yield and count by protecting islet cells from the oxidative stress created during islet isolation through increasing GCLC and NQO1.

### 3.2. DMF Administration Retarded/Prevented the Onset of Autoimmune Diabetes in NOD Mice

**Figure 3:**
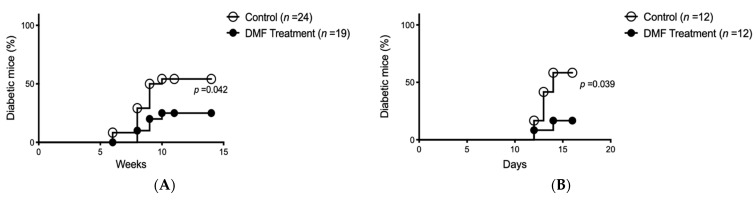
DMF treatment reduces diabetes onset in spontaneous and accelerated non-obese diabetic (NOD) diabetes model. (**A**) Spontaneous diabetes onset in NOD mice was significantly decreased in the DMF-treated group compared to the control group (*p* = 0.042) at 14 weeks. (**B**) In cyclophosphamide-induced accelerated NOD diabetes model, DMF treatment significantly decreased the incidence of diabetes compared to the control group (*p* = 0.039) at 2 weeks.

Since the antioxidative effects of DMF on islet cells were confirmed with the rat isolation model, the NOD autoimmune diabetes model was used for further study. Spontaneous autoimmune diabetes occurred in 54.2% of the control NOD mice (*n* = 24) at a median age of 16 weeks (range, 14–18 weeks). The onset of diabetes was significantly reduced to 25% (*n* = 19; median, 17 weeks; range, 16–18 weeks of age) in the DMF-treated group (Figure 3A). At 22 weeks, DMF administration was discontinued in the DMF-treated normoglycemic mice to confirm the effects of DMF on the prevention of diabetes. Hyperglycemia (diabetes) was observed in 66.7% of the previously DMF-treated group within another 6 weeks (*n* = 6; median, 25.5 weeks; range, 25–26 weeks of age). On the other hand, DMF administration was initiated in the DMF-untreated hyperglycemic NOD mice to investigate if DMF could reverse autoimmune diabetes. DMF administration in the diabetic NOD mice could not reverse or improve hyperglycemia within 6 weeks (*n* = 6).

### 3.3. DMF Administration Prevented Accelerated Autoimmune Diabetes Onset in NOD Mice

The accelerated autoimmune diabetes model was used to further investigate how DMF prevented hyperglycemia in NOD mice. 8-week-old female NOD mice were given a single dose of cyclophosphamide (200 mg/kg) intraperitoneally after the confirmation of no hyperglycemia. In the DMF-treated group, DMF was given to the mice (25 mg/kg) by oral gavage 12 and 24 h prior to cyclophosphamide administration, and continued twice daily for 2 weeks. The onset of diabetes in 58.3% of the DMF-untreated mice (*n* = 12) within a median of 13 days was observed. DMF treatment significantly reduced the incidence of diabetes to 16.7% (*n* = 12; median, 13 days; range, 12–14 weeks of age) (Figure 3B).

### 3.4. DMF Attenuated Insulitis and Decreased Serum Cytokine Levels in NOD Mice

**Figure 4:**
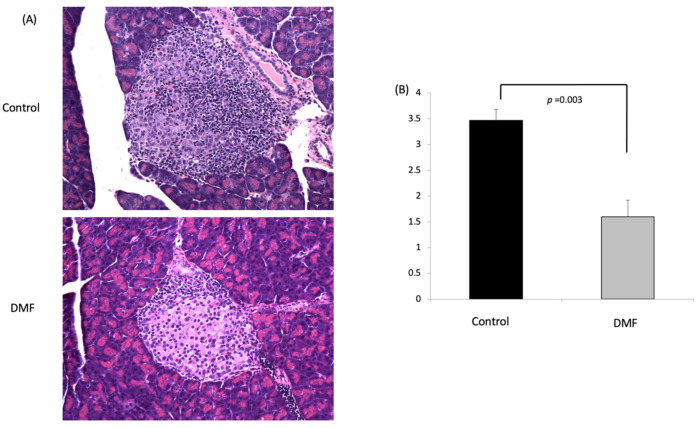
DMF treatment protects pancreatic islets and reduces insulitis in accelerated model of NOD mice. (**A**) Representative images of H&E-stained pancreatic section from DMF-treated group and control group; magnification: x200. Compared to DMF-treated group, severe lymphocyte infiltration in the islets was found in control group. (**B**) DMF treatment significantly reduced insulitis score compared to control group (*n* = 4).

Two weeks after cyclophosphamide injection, all the animals were euthanized for tissue and blood harvest. In the control group, most of the islets exhibited severe insulitis, while the DMF-treated group had mild insulitis, and most of their islets remained intact (Figure 4A). The insulitis score in the DMF group was significantly lower when compared to the control group (1.6 ± 0.32 vs. 3.47 ± 0.21, respectively, *p* < 0.05) (Figure 4B).

**Figure 5:**
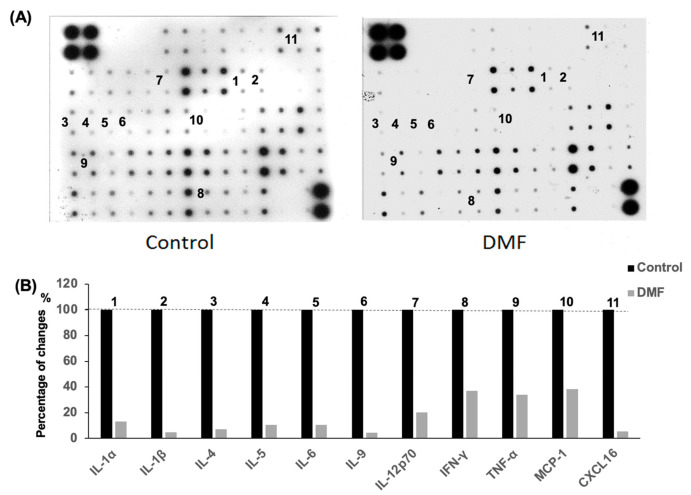
Impact of DMF treatment on serum cytokine levels in accelerated NOD mouse model. (**A**) The systemic inflammatory state was assessed using the mouse cytokine and chemokine array assay. Sera from five mice in each group were used for the assay. There were duplicated spots for each cytokine or chemokine. (**B**) Relative expression in the DMF group was calculated using control group as the benchmark. The levels of cytokines and chemokines including IL-1α, IL-1β, IL-2, IL-4, IL-5, IL-6, IL-9, IL-12p70, IFN-γ, TNF-α, MCP-1 and CXCL16 were lower in DMF-treated compared to control group (*n* = 5 for each group).

The inflammatory cells that infiltrate islet cells are well known to secrete a mixture of proinflammatory cytokines such as TNF-α, IL-6 and IL-1β, which eventually promote β-cell destruction. The mouse cytokine antibody array was used to evaluate the serum cytokine levels in the accelerated diabetes model. The serum levels of the proinflammatory cytokines and chemokines IL-1α, IL-1β, IL-2, IL-4, IL-5, IL-6, IL-9, IL-12p70, IFN-γ, TNF-α, MCP-1 and CXCL16 in the DMF-treated group were lower than those found in the control group at day 14 after cyclophosphamide administration (Figure 5).

Our data suggested that the lower levels of proinflammatory cytokines and chemokines in the serum in the DMF-treated group might contribute to the attenuation of insulitis and the lower rate of diabetes onset.

## 4. Discussion

Oxidative stress and its companion, inflammation, play a central role in the pathogenesis and progression of numerous diseases. Importantly, both experimental and clinical studies have demonstrated the role of oxidative stress in the pathogenesis of type 1 diabetes [21,22]. Increased ROS production and an impaired antioxidant defense system represent the main cause of oxidative stress. Nrf2, a transcription factor, regulates the expression of antioxidant and anti-inflammatory genes, and its impaired activity plays a key role in the pathogenesis of oxidative stress. Several studies have demonstrated that based on its antioxidant and anti-inflammatory properties, the Nrf2 pathway represents an attractive target for treating several chronic autoimmune diseases [23,24]. 

Our previous study [10] demonstrated that the Nrf2 pathway plays a significant role in protecting islets against the oxidative stress created during the islet isolation process, using Nrf2-deficient mice. We also confirmed that the pharmacological activation of the pathway by an Nrf2 activator, dh404, was able to protect islets against oxidative stress and significantly improve islet yield, viability and β-cell content. The therapeutic potential of another Nrf2 pathway activator, DMF, was evaluated in this study. DMF is a highly lipophilic, mobile methyl ester, which, by activating the Nrf2 pathway, has been shown to reduce the relapse rate, slow the progression of multiple sclerosis [25,26,27], and alleviate chemical-induced colonic inflammatory damage [28,29]. However, the exact mechanism of action of DMF is yet to be clarified. Hu et al. reported that DMF could potentially prevent diabetes-induced myocardial tissue injury through Nrf2 pathway activation [12]. We also reported that DMF protected human pancreatic islet cells from oxidative stress by upregulating the expression of antioxidant molecules in vitro and rat islets in vivo from L-arginine-induced chronic pancreatitis [14]. In the current study, we therefore used the rat islet isolation method first to investigate the cytoprotective effects of DMF on islet cells during the pancreatic islet isolation process. The islet isolation process consists of mechanical disruption, chemical digestion, and warm and cold ischemia events and is well known to cause severe oxidative stress to the islet cells during the process.

As expected, DMF administration significantly improved both the islet yield and count by increasing the self-defense system in the islet cells against the stress induced by the pancreatic islet isolation process. This finding was consistent with the result of our previous study using another Nrf2 activator, RTA dh404 [10]. DMF may have the potential to compensate for the low β-cell antioxidant capacity, which is the main cause of vulnerability to oxidative stress [9].

Under oxidative stress, Nrf2 dissociates from Keap1 and translocates into the nucleus. In the nucleus, Nrf2 binds to its specific target genes, which results in the increased transcription of phase II antioxidant enzymes. GCLC and NQO1 are key phase II enzymes that play an important role in redox homeostasis, protecting against oxidative stress, and cellular defense. Some studies have demonstrated that GCLC and NQO1 play a key role in the protection of islets in a streptozotocin-induced diabetic rat model [30,31]. Importantly, by using isolated rat islet cells, we were able to investigate how DMF administration regulated the mRNA expression of GCLC and NQO1 in the islet cells. The mRNA expression of GCLC and NQO1 in isolated islets was significantly increased by DMF administration. In addition, we evaluated the expression of 8-OHdG, a common biomarker for oxidative DNA damage, in the isolated islet cells. A study by Ku Y.P. et al. showed that 8-OHdG is a sensitive and reliable biomarker for evaluating streptozotocin-induced oxidative DNA damage in islet cells [32]. In this study, DMF administration significantly reduced 8-OHdG-positive cells in isolated islets. Taken together, our results demonstrate that DMF was able to protect pancreatic islet cells against oxidant stress by increasing the expression of GCLC and NQO1 genes during the islet isolation process. Based on these findings, we hypothesized that DMF administration may protect islet cells from ROS, leading to the prevention or delaying of the onset of autoimmune diabetes in NOD mouse via its strong antioxidant and anti-inflammatory properties.

Diabetes in the NOD mouse is characterized by the infiltration of inflammatory cells into the pancreas, resulting in the T cell-mediated destruction of β-cells. The process is very similar to the pathogenesis of type I diabetes in humans. For over 30 years, the NOD mouse has been used as an important and valuable tool in exploring the mechanisms and treatment of diabetes [33]. In this study, we used both spontaneous and accelerated diabetes models of the NOD mouse to explore the effect of DMF in preventing or delaying the onset of type 1 diabetes. In the spontaneous diabetes model, the administration of DMF significantly decreased the onset of diabetes from 54.2% to 25%. Furthermore, among the treated mice that developed diabetes, DMF delayed the onset of the disease from 16 weeks in the control group to 17 weeks in the treated group. In order to confirm the role of DMF in preventing and delaying the onset of diabetes, we discontinued the administration of DMF at week 22. Within 6 weeks after the discontinuation of DMF, 4 out of the 6 mice (66.7%) became diabetic in this group. Additionally, to determine if DMF administration could reverse or improve diabetes after the disease onset, we started to treat the diabetic NOD mice at week 22 with the same dose of DMF for six weeks; DMF administration failed to attenuate or reverse diabetes in the NOD mice with established diabetes. Taken together, these findings demonstrate the efficacy of DMF in preventing or delaying the onset and progression of the disease at the early stage of the onset of diabetes but not in the treatment of established diabetes. However, a limitation of this study is that the effect of the administration of DMF in diabetic NOD mice was only observed for 6 weeks. Further research focusing on β-cell proliferation or regeneration will be needed to evaluate long-term effects of DMF on diabetic NOD mice.

An accelerated diabetes model was used to investigate how DMF could prevent or delay hyperglycemia in NOD mice. We used a higher dose of DMF (25 mg/kg, twice daily) in the accelerated diabetes model. The administration of DMF significantly reduced the incidence of diabetes from 58.3% to 16.7%. Histology clearly demonstrated that DMF significantly attenuated insulitis.

It is well known that inflammatory cell infiltration into the pancreas leads to the production of inflammatory cytokines and ROS, which eventually cause permanent β-cell destruction in patients with type I diabetes. We investigated the hypothesis that the anti-inflammatory effect of DMF could minimize insulitis by reducing circulating cytokine and chemokine levels. We found a significant reduction of circulating proinflammatory cytokines and chemokines including IL-1α, IL1-β, IL4, IL5, IL6, IL9, IL12p70, IFN-γ, TNF-α, MCP-1 and CXCL16 in the DMF-treated group. Many cytokines and chemokines have been shown to cause detrimental damage to islet cells. IL1-β in combination with IFN-γ and TNF-α plays an important role in β-cell dysfunction and death [34,35]. Those cytokines were produced from not only inflammatory cells but also islets and induced apoptosis in human islets [36,37,38]. It has been reported that IL-12 impaired glucose-stimulated insulin secretion and induced IFN-γ production in INS-1 cells and human islet cells. IL-12 also plays critical roles in producing immune responses and regulating the functions of a variety of inflammatory cells and, therefore, is an important therapeutic target in many inflammatory diseases including the CNS autoimmune diseases, uveitis and multiple sclerosis [39]. The production of MCP-1 by islet cells contributes to the recruitment of monocytes into islet cells in early type 1 diabetes [40]. Piemonti L. et al. also reported that lower MCP-1 production from human islet preparations was associated with a better clinical outcome in islet transplantation for patients with type I diabetes [41]. Moreover, CD11c^+^ cells in islets produce many chemokines that recruit T cells to the islets, and CXCL16 is one of the highest expressed chemokines in islet CD11c^+^ cells in NOD mice [42]. Our data suggest that DMF may prevent/attenuate insulitis and β-cell apoptosis by reducing proinflammatory cytokine and chemokine levels in the serum as well as preventing inflammatory cell infiltration into the pancreas. However, more specific and sensitive cytokine measurements such as ELISA and intracellular cytokine and chemokine staining in infiltrating inflammatory cells are needed to further investigate the regulation of anti-inflammatory impacts by DMF in the NOD mouse model.

A study by Wu Q et al. demonstrated that DMF selectively reduces T cell activation in patients with multiple sclerosis. DMF treatment most significantly decreased the proinflammatory Th1 and Th17 subsets, while increasing the anti-inflammatory Th2 subset [43]. Moreover, a recent study by Schultheis et al. reported that Nrf2 activators protect β-cells against glucolipotoxicity by preserving mitochondrial function and redox balance [44]. Further studies are needed to explore the underlying mechanisms of the cytoprotective effects of DMF in autoimmune diabetes.

The potential benefits of DMF have been investigated in other diseases caused by severe inflammation and oxidative stress, too. DMF has already been approved for the treatment of patients with multiple sclerosis [27,45] and psoriasis [46,47]. Clinical trials for other diseases have been ongoing, including amyotrophic lateral sclerosis [48] and cutaneous T cell lymphoma [49]. Many researchers have also reported beneficial effects of DMF on other diseases or medical conditions caused by severe inflammation and oxidative stress such as colitis [28,50], Parkinson’s disease [51,52], melanoma [53,54,55], breast cancer [56], lung fibrosis [57], pancreatitis [14,20], liver injury [15,58], intracerebral hemorrhage [59] and sickle cell disease [60] through preclinical animal studies. Diabetes with chronic hyperglycemia is a systemic chronic disease and creates oxidative stress across the entire body, which significantly increases the risks for many health problems including the development of retinopathy, nephropathy, neuropathy and cardiomyopathy [61]. DMF may also protect patients from the exacerbation of diabetic complications. Thus, the administration of DMF may become an important asset in the management of type 1 diabetes.

## 5. Conclusions

In summary, DMF significantly reduced the incidence of autoimmune diabetes in NOD mice by (1) promoting the self-defense system in islet cells, protecting against oxidative stress through increasing GCLC and NQO1, and (2) reducing the islet insults caused by proinflammatory cytokines and chemokines that are mainly produced by inflammatory cells infiltrating islet cells. Our findings may be of assistance in the future repurposing of DMF in the management of type I diabetes.

## Data Availability

The authors confirm that the data supporting the findings of this study are available within the article.

## Abbreviations

Nrf2: nuclear factor erythroid-derived 2-related factor;
DMF: Total phenolic content;
GCLC: Total flavonoid content;
NQO1: NAD(P)H quinone dehydrogenase 1;
Nrf2: nuclear factor erythroid-derived 2-related factor;
ROS: reactive oxygen species;
SD: standard deviation;
NOD: non-obese diabetic mice.
